# The effects of testosterone replacement therapy on the prostate: a clinical perspective

**DOI:** 10.12688/f1000research.16497.1

**Published:** 2019-02-25

**Authors:** Saiful Miah, Tharu Tharakan, Kylie A Gallagher, Taimur T Shah, Mathias Winkler, Channa N Jayasena, Hashim U Ahmed, Suks Minhas

**Affiliations:** 1 Department of Urology, Imperial College Healthcare NHS Trust, Charing Cross Hospital, Fulham Palace Road, London, W6 8RF, UK; 2 Division of Surgery and Interventional Science, University College London Medical School, 21 University Street, London, WC1E 6AU, UK; 3 Division of Surgery, Department of Surgery and Cancer, Faculty of Medicine, Imperial College London, London, SW7 2AZ, UK; 4 Section of Investigative Medicine, Department of Medicine, Imperial College London, London, W12 0NN, UK

**Keywords:** Prostate cancer, testosterone replacement therapy, late onset hypogonadism, androgen deprivation, andropause

## Abstract

Male hypogonadism is a clinical syndrome characterized by low testosterone and symptoms of androgen deficiency. Prostate cancer remains a significant health burden and cause of male mortality worldwide. The use of testosterone replacement therapy drugs is rising year-on-year for the treatment of androgen deficiency and has reached global proportions. As clinicians, we must be well versed and provide appropriate counseling for men prior to the commencement of testosterone replacement therapy. This review summarizes the current clinical and basic science evidence in relation to this commonly encountered clinical scenario. There is gathering evidence that suggests, from an oncological perspective, that it is safe to commence testosterone replacement therapy for men who have a combination of biochemically confirmed androgen deficiency and who have either had definitive treatment of their prostate cancer or no previous history of this disease. However, patients must be made aware and cautioned that there is a distinct lack of level 1 evidence. Calls for such studies have been made throughout the urological and andrological community to provide a definitive answer. For those with a diagnosis of prostate cancer that remains untreated, there is a sparsity of evidence and therefore clinicians are “pushing the limits” of safety when considering the commencement of testosterone replacement therapy.

## Introduction

Male hypogonadism is a clinical syndrome attributed to androgen deficiency (AD). It can detrimentally affect multiple organ functions in addition to having detrimental effects on quality of life in men. Testosterone levels progressively decrease with age ^1^. After the age of 40 years, testosterone levels in men have been shown to decrease 1 to 2% per year ^2– 4^. By the seventh decade, 35% of men have been shown to have lower testosterone levels than younger men ^5^. This has led to the emergence of a group of men older than age 65 years with hypogonadism with so-called “late-onset” hypogonadism (LOH) with its extensive symptom complex including obesity and loss of libido ( Table 1).

**Table 1.** “Late-onset” hypogonadism symptom complex.

Loss of libido Erectile dysfunction Overweight or obesity Sarcopenia Low bone mineral mass/density Negative mental health impacts, including depression Fatigue Loss of body hair Hot flashes Loss of vigor and frailty Depressive symptoms Poor memory/concentration Reduced lean muscle mass

Prostate cancer (PCa) remains a significant health burden and cause of male mortality. The National Cancer Institute has estimated that, in the US, over 3 million men are living with PCa ^6^. It was also estimated that an additional 164,690 cases would be diagnosed in the US during 2018 alone ^6^. The seminal and Nobel prize–winning study by Huggins and Hodges established the androgen hypothesis in that the growth and progression of PCa are directly related to male androgenic activity when withdrawing these hormones ^7^. However, nearly 80 years after this publication, there remains controversy and great debate on whether exogenous administration of testosterone will increase the risk of development or the progression of PCa ^8, 9^. The urgent need for an answer to this is due in part to the sheer volume of the administration of testosterone replacement therapy (TRT), which is now one of the most widely prescribed medications in the US ^10^. The scientific community still seeks a definitive stance on the oncogenic safety profile of TRT in relation to PCa. In this review, we address the issue of whether there is an association between TRT and PCa by using evidence ranging from basic science to genetic and major epidemiological studies conducted on this topic.

## Pathophysiology of androgen-driven prostate cancer

In 1941, Huggins and Hodges demonstrated that testosterone was an oncogenic driving hormone in PCa ^7, 11^. In their study, acid phosphatase activity was elevated in serum specimens of 21 out of 47 PCa cases and 19 out of 25 cases with confirmed bone metastasis ^7, 11^. Eight men who harbored skeletal metastasis then underwent castration ^7, 11^. Their elevated acid phosphatase levels fell rapidly, indicating the androgen dependence of this oncological biomarker ^7, 11^. The subsequent administration of testosterone to these men restored acid phosphatase to a level higher than pre-castration levels ^7, 11^.

Owing to their extensive first-pass metabolism through the liver, oral preparations of testosterone are not widely used in TRT ^12^. Methods of replacement are administered predominantly by transdermal gel or intramuscular depot injection. Testosterone is metabolized to 5α- dihydrotestosterone (DHT), and both testosterone and 5α-DHT contribute to prostatic growth ^12^. There is abundant evidence that, within an *in vitro* environment, PCa cell lines are androgen-dependent in that they will undergo apoptosis and reduced cellular proliferation with the withdrawal of this hormone ^13, 14^. For cell lines, androgens are critical for the development and maintenance of normal and cancer tissue, and the androgen receptor (AR) is the main therapeutic target for PCa ^13^. This phenomenon is further supported by *in vivo* studies where the administration of androgens creates a pro-tumorogenic environment in murine xenograft models, and the converse effect follows androgen suppression ^15^.

Androgen deprivation therapy (ADT) using gonadotrophin-releasing hormone (GnRH) super-agonists remains the cornerstone of the treatment of metastatic PCa. ADT for the majority of PCas will progress to androgen-independent disease ^16^. The development of resistance to apoptosis associated with androgen independence is one of the critical later stages of the molecular hallmarks of advanced PCa ^17^. Several mechanisms have been postulated to explain the transformation of ADT-responsive PCa to castrate-resistant PCa. These include overexpression of the AR, mutations in the AR, altered recruitment of transcription cofactors, and sustained intratumoral synthesis of DHT ^18^.

## Androgen saturation theory

The androgen saturation theory proposes that the effects of testosterone on the prostate are limited by the capacity and concentration of prostate ARs ^19^. Therefore, testosterone above a given threshold level will have no effect on the prostate ^19^. In prostatic tissue, the AR becomes saturated at about 4 nmol/L (120 ng/dL) *in vitro*, corresponding to about 8 nmol/L (240 ng/dL) *in vivo* because of the presence of binding hormones ^20, 21^. This model suggests why serum testosterone may appear unrelated to PCa risk in the general population and why testosterone administration in men with metastatic PCa causes rapid progression in castrated but not hormonally intact men ^19^. This is a significant shift of paradigm from the historical dogma that testosterone drives PCa ^22^.

Clinically, this theory was consolidated with the discovery that, although TRT increases serum testosterone levels, it did not correlate with intraprostatic testosterone levels on biopsy specimens of men on TRT ^23^. Furthermore, in-depth proteomic and genomic analysis of these histological specimens failed to demonstrate an increased expression of PCa-related tissue biomarkers ( *AR, Ki-67*, and *CD34*) or genes ( *AR, prostate-specific antigen [PSA], PAP2A, VEGF, NXK3,* and *CLU [Clusterin*]) ^23^.

## Testosterone replacement therapy in men without a diagnosis of prostate cancer

The majority of studies on TRT and PCa risk are limited by either insufficient power to determine this risk or their retrospective design ( Table 2) ^24^. In 2005, Calof *et al*. ^25^ reported a meta-analysis of 19 studies, which included 651 men who received TRT, demonstrating that there was no statistically significant difference in PCa diagnoses among those who used testosterone ^25^. Haider *et al*. ^26^ reported on a multi-center prospective cohort study consisting of a total of 1023 hypogonadal men who received TRT. With a median follow-up of 5 years, the incidence of PCa was lower in TRT-treated populations than accepted incidence rates from large population-based studies with long-term follow-up ^24, 26^. The limitations of this study include a younger cohort of patients with a mean age of only 58 years, thus making it challenging to draw comparisons with large screening trials such as Prostate, Lung, Colorectal, and Ovarian (PLCO) and the European Randomized Study of Screening for Prostate Cancer ^27^. For those men who harbor high-grade prostatic intraepithelial neoplasia (PIN), it has been shown that with short-term follow-up there is no greater risk of an increase of their PSA or PCa compared to those men without PIN ^28^.

**Table 2.** Selected studies which support the absence of a link between testosterone replacement therapy and prostate cancer.

Authors	Year	Study format	Number on testosterone replacement therapy (TRT)	Conclusions
Loeb *et al*. ^29^	2017	Nested case-control	1,662	No overall increase in risk of prostate cancer (PCa). Early increase in the risk of favorable cancer (odds ratio [OR] 1.35, 95% confidence interval [CI] 1.16–1.56) and decrease in the risk of aggressive PCa (OR 0.50, 95% CI 0.37–0.67).
Haider *et al*. ^26^	2015	Prospective cohort (multi-center)	1,023	Lower incidence of PCa in TRT-treated populations
Eisenberg *et al*. ^33^	2015	Retrospective observational	247	There was no change in cancer risk overall, or PCa risk specifically, for men older than 40 years using long-term TRT.
Feneley *et al*. ^34^	2012	Prospective cohort (single-center)	1,365	Incidence of PCa during long-term TRT was equivalent to that of the general population.
Calof *et al*. ^25^	2005	Meta-analysis	651	No statistically significant difference in PCa diagnoses among TRT users
Rhoden and Morgentaler ^28^	2003	Prospective cohort (single-center)	75	After 1 year of TRT, men with prostatic intraepithelial neoplasia (PIN) do not have a greater increase in prostate-specific antigen or a significantly increased risk of cancer than men without PIN.

## Contemporary epidemiological studies

A recently published article by Loeb *et al*. ^29^ reported a large nested case-control study which included over 38,000 and 192, 000 men with diagnosed PCa or free from the disease, respectively. This study demonstrated that there was no overall increase in risk of PCa in those men who received TRT (odds ratio [OR] 1.03, 95% confidence interval [CI] 0.90–1.17). Once the breakdown of disease subtype was analyzed between favorable (T1–T2, PSA <10 ng/mL, Gleason score [GS] ≤6, not N1, no M1) and aggressive (local high risk, T1–T2, GS of 8–10, PSA 20–50 ng/mL, no N1, no M1) disease, locally advanced (T3, PSA >50 ng/mL, not N1, not M1), regionally metastatic (T4, PSA 50–100 ng/mL, N1, no M1), and metastatic (metastases on bone imaging or PSA >100 ng/mL) results demonstrated the clinical benefits of commencing TRT. TRT demonstrated an early increase in favorable-risk PCa (OR 1.35, 95% CI 1.16–1.56), which was balanced with a finding that those men on TRT significantly lowered their risk of harboring aggressive PCas (OR 0.50, 95% CI 0.37–0.67). The vast majority of favorable PCa fell within the spectrum of clinically insignificant PCa ^30^. There has been a recent shift and drive to change the nomenclature associated with PCa, including calls to drop the label “cancer” with certain favorable-risk PCa ^30^. Well-characterized pure Gleason grade 3 disease fails to clearly show both clinical and molecular hallmarks that are expected of a cancer. Hence, a strategy that decreases the incidence of aggressive PCa at the expense of its favorable-risk counterpart would warrant further investigation and would be welcomed if robustly proven.

## Testosterone replacement therapy with treated prostate cancer

There is now gathering evidence that men who have had their PCa treated by radical prostatectomy (RP) and external beam radiotherapy with curative intent are potentially safe to have TRT administered. However, again, these studies have inherent limitations, including the limited number of patients, the lack of long-term follow-up, and the fact that the study was designed to be a retrospective case series.

Kaufman and Graydon reported on seven men who underwent RP and who then were placed on TRT for biochemically confirmed hypogonadism ^31^. On follow-up of these surgically treated men, no one was found to have either biochemical or clinical recurrence of their malignancy ^31^. Khera *et al*. ^32^ reported on a significantly larger cohort of men (n = 57) who underwent RP with confirmed negative surgical margins and subsequently commenced on TRT. With an average follow-up of 13 months (range of 1–99 months), no men in their study demonstrated biochemical recurrence of their cancer with their regular PSA surveillance ^32^. Similarly, for those men who opted to undergo external beam radiotherapy for their PCa and then commenced on TRT, Pastuszak *et al*. ^35^ concluded that there was only a minor increase in serum PSA and a low rate of biochemical recurrence in their multi-center series of 98 men ^35^. The authors also commented that their 6.1% biochemical recurrence was lower than previously reported rates for radiation therapy, suggesting that TRT does not lead to biochemical failure in those men who received radical radiotherapy for their PCa ^35^.

## Testosterone replacement therapy with untreated prostate cancer

A recent systematic review by Kaplan *et al*. ^36^ highlighted the caution required when commencing TRT for men undergoing active surveillance (AS) or watchful waiting of their PCa as this is “pushing the limits of safety”. This conclusion was based primarily on the sparsity of evidence in this cohort of men with very minimal reports in comparison with those with no diagnosis or treated PCa. Kacker *et al*. ^37^ recently reported their retrospective study in 28 men on AS for their PCa who commenced TRT for AD in comparison with 96 men in the untreated AD arm. The authors reported that biopsy progression rates were similar between these two groups over a 3-year follow-up period and appear unaffected by TRT ^37^.

## Non-oncological consequences of androgen deficiency

The metabolic syndrome (MetS) was first described in 1923 by Kylin. Later, Reaven coined the term “Syndrome X”, which is a constellation of insulin resistance, hyperglycemia, hypertension, low high-density lipoprotein cholesterol, and increased very-low-density lipoprotein and triglyceride to further define MetS ^38^. Owing to its associated risk of cardiovascular disease, MetS has been proposed as the main threat to public health in the 21st century ^39^. There are several definitions of MetS; however, the primary emphasis is on central obesity ( Table 3), and the estimated prevalence of this among males in the US is 34.5% ^40, 41^.

**Table 3.** Definition of metabolic syndrome ^41^.

Central abdominal obesity waist circumference of at least 94 cm in Europids and of more than 90 cm in Asians AND	
2 out of 4	elevated triglycerides ≥1.7 mmol/L (≥150 mg/dL)
	reduced high-density lipoprotein cholesterol <1.03 mmol/L (<40 mg/dL)
	elevated systolic blood pressure ≥130 mm Hg, diastolic blood pressure ≥85 mm Hg (or treatment)
	dysglycemia (raised fasting plasma glucose, fasting blood glucose ≥5.6 mmol/L [≥100 mg/dL]) (or type 2 diabetes mellitus)

There is a strong inverse relationship with testosterone and body fat in men, and numerous epidemiological studies show the increase of MetS with declining testosterone levels ^42, 43^. The central fat seen with abdominal obesity has high aromatase activity converting testosterone to estradiol ^43^. Furthermore, testosterone promotes lipolysis and inhibits adipocyte development ^43^.

About 50% of men with diagnosed PCa will be exposed to ADT at some stage of their disease ^44^. Again, the clinical adverse effects of low testosterone due to ADT are well reported and have a negative effect on quality of life ^45, 46^. ADT in those with PCa has been linked to a metabolic-type syndrome of insulin insensitivity with its associated central obesity and decreased muscle mass ^45^.

Numerous interventional studies have shown that TRT in hypogonadal men with MetS has beneficial effects on central adiposity, insulin resistance, and glycemic control ^43, 47^. In addition, TRT has shown improvements in well-established cardiovascular risk factors by lowering elevated blood pressure, triglyceride levels, and cholesterol ^43^. TRT has also been shown, in a prospective manner, to address the detrimental sexual dysfunction associated with LOH ^48^. The administration or TRT has been shown to result in a significant improvement in the various aspects of sexual function, including sexual desire, intercourse satisfaction, and overall satisfaction ^48^. Similarly, TRT has demonstrated significant improvement in depressive symptoms in those who received treatment within the setting of a randomized trial ^49^.

## Commercial caution

TRT drugs have been a phenomenal commercial success and their pharmaceutical sales are rising year-on-year ^10^. The off-label indications have led to an exponential increase in the prescription of TRT in the US, and commercial marketing efforts are potentially linked to prescriber habits ^50^. This increased commercialism had included the use of TRT without the clinical or biochemical confirmation of hypogonadism, and there are calls to limit overtreatment with the application of strict diagnostic criteria of LOH ^50^. Alarmingly, there has been a fourfold increase in the use TRT in the younger cohort of men (18–45 years) in the US ^51^. This has potential clinical implications—not the least of which is the potential detrimental effect on fertility in this age group—that may not manifest until after several decades. This increased use in younger men would require future focused studies assessing the long-term oncological sequelae of early TRT use. Crucially, the potential oncological manifestations of TRT use in younger men may not reveal themselves for several decades.

Testosterone dependence has also been identified ^52^. The cessation of TRT ultimately will result in the restoration of baseline serum testosterone levels; however, these men may feel markedly symptomatic and seek additional testosterone while waiting for normal levels to be achieved ^52^. The current European Association of Urology guidelines on male hypogonadism advocate the use of TRT, including in those adult men with consistent and preferably multiple signs and symptoms of hypogonadism and a low testosterone following unsuccessful treatment of obesity and comorbidities.

## Conclusions

With the increased use of TRT for the treatment of AD, it is essential that the clinician be well versed in its potential oncological implications. At present, there is no definitive evidence that administration of exogenous testosterone will increase the incidence of PCa. The absence of a large randomized clinical trial to address this topic is starkly obvious; such a study is vital before we can confidently counsel men who express interest in or harbor a clinical need for TRT. At best, we can provide these men only sub–level 1 evidence of TRT in relation to PCa, highlighting the ambiguity and dearth of high-quality studies. In our clinical practice, when prescribing TRT, we make it paramount that patients be made aware that, although there may be a significant improvement in the signs and symptoms of LOH, the unlikely risk of developing a significant *de novo* or recurrence of their treated PCa as a direct result of pharmacological intervention is currently based on less-than-optimal data. When we are faced with those men with untreated PCa or on AS, we avoid TRT at present. However, we eagerly await further evidence that would suggest otherwise.
